# An insight of anopheline larvicidal mechanism of *Trichoderma asperellum* (TaspSKGN2)

**DOI:** 10.1038/s41598-021-95310-1

**Published:** 2021-08-06

**Authors:** Swapan Kumar Ghosh, Dipanjan Podder, Anirvan Mukherjee

**Affiliations:** Molecular Mycopathology Lab, Biocontrol and Cancer Research Unit, PG Department of Botany, Ramakrishna Mission Vivekananda Centenary College (Autonomous), Rahara, Kolkata, 700118 India

**Keywords:** Microbiology, Zoology, Ecology, Environmental sciences

## Abstract

Anopheline larvicidal property of *T. asperellum* has been found recently in medical science. The mechanism of actions exhibited by *T. asperellum* to infect mosquito larvae is the pivotal context of our present study. To infect an insect, entomopathogens must undergo some events of pathogenesis. We performed some experiments to find out the mechanisms of action of *T. asperellum* against anopheline larvae and compared its actions with other two well recognized entomopathogens like *Metarhizium anisopliae* and *Beauveria bassiana.* The methodology adopted for this includes Compound light and SE Microscopic study of host–pathogen interaction, detection of fungal spore adhesion on larval surface (Mucilage assay), detection of cuticle degrading enzymes (Spore bound pr1, chitinase and protease) by spectro-photometric method, Quantitative estimation of chitinase and protease enzymes, and determination of nuclear degeneration of hemocyte cells of ME (methanolic extract) treated larvae by *T. asperellum* under fluorescence microscope. Compound light microscopic studies showed spore attachment, appressorium and germ tube formation, invasion and proliferated hyphal growth of *T. asperellum* on epicuticle and inside of dead larvae. SEM study also supported them. After 3 h of interaction, spores were found to be attached on larval surface exhibiting pink colored outer layer at the site of attachment indicating the presence of mucilage surrounding the attached spores. The enzymatic cleavage of the 4-nitroanilide substrate yields 4-nitroaniline which indicates the presence of spore-bound PR1 protein (Pathogenecity Related 1 Protein) and it was highest (absorbance 1.298 ± 0.002) for *T. asperellum* in comparison with control and other two entomopathogens. *T. asperellum* exhibited highest enzymatic index values for both chitinase (5.20) and protease (2.77) among three entomopathogens. Quantitative experiment showed that chitinase enzyme concentration of *T. asperellum* (245 µg mL^−1^) was better than other two *M. anisopliae* (134.59 µg mL^−1^) and *B. bassiana* (128.65 µg mL^−1^). Similarly protease enzyme concentration of this fungus was best (298.652 µg mL^−1^) among three entomopathogens. Here we have detected and estimated fragmentized nuclei of hemocyte cells by fluorescence microscopy in treated larvae with different ME doses of *T. asperellum,* and also observed that mosquito larvae exposed to 0.1 mg mL^−1^ dose of ME showed maximum (100%) nuclear fragmentations of hemocytes and while 20, 45, 70 and 85% of nuclear deformities were recorded at 0.02, 0.04, 0.06 and 0.08 mg mL^−1^ concentrations of ME. The knowledge of this work certainly will help in understanding of mechanism of action of *T. asperellum* for anopheline larval killing and consequently in eradication of malaria vector.

## Introduction

Of late/recently the world is going through an extremely challenging and tough period as it is severely affected by several deadly mosquito borne diseases like Malaria, Dengue, Chikunguniya, etc. A thorough knowledge of the physico-chemical factors, which influence mosquito habitat on larval production, and a good understanding of the biological and ecological aspects of mosquito vector species are of great importance in the case of formulating effective plan and careful implementation of integrated vector control strategies by environmental management^1^. Recently Ghosh et al.^1^ reported that the adult mosquito aquatic niches parameters have a great role for the integrated mosquito control programme. On the other hand, chemical insecticides are randomly used to keep mosquitoes in control, but they have hazardous effects on environment and human health. So, bio-insecticides along with mosquito’s larval aquatic niches can be a good alternative for chemical insecticides^2^. The survey, description and application of insect pathogens are globally important^3^. Bio control agents like *B. bassiana* and *M. anisopliae* have been used for several years to fight insects by many workers^4–7^, as they are natural enemies of agricultural pests and have a great role in maintaining ecological balance^8,9^. But entomopathogenecity of *Trichoderma longibrachiatum* and *Trichoderma asperellum* has been established in our laboratory as first reports in previous studies^10,11^. *T. asperellum* is a well known fungus in agriculture. It is regularly used in agriculture for many years both as bio control agents to curb plant pathogenic microbes and plant growth promoting agents, but anopheline larvicidal efficacy of *T. asperellum* (T.aspSKGN2) (GenBank Accession No. MG719999.1) is novel. The mechanisms of action exhibited by *T. asperellum* to infect mosquito larvae is the pivotal context of our present study. To infect an insect, entomopathogens must undergo some infection processes. To begin infection processes, at first fungal spores have to attach to the host surface by secreting adhesives^12^. Detection of fungal mucilage for attachment of spore to host surface is an important criterion to experiment fungal mechanism of infection. After successful attachment of spore, fungi have to invade insect cuticle either by mechanical process or enzymatic degradation, through formation of appresorium and then infection peg^13^. The main components of insect cuticle are chitin and other proteins^14^. Virulence of fungal pathogens can be determined by assaying enzymatic activities related to infection pathways, such as spore bound protease (Pr1), chitinases^15,16^, etc. After penetrating the host body through invasion of cuticle, the entomopathogens secrete some toxic compounds inside haemocoel or other tissues of the larval body^17^. Toxic compounds may reduce the insect Phenol Oxidase (PO) content and degenerate insect hemocytes, causing loss of insect immunity^18^. Study of cytotoxicity to insect immune cells is a parameter of assaying insecticidal efficacy. Although the mechanisms of entomopathogenecity of other entomopathogenic fungi are well known to us, the mechanism of novel entomopathogen *T. asperellum*, as reported by us previously^11^, has hardly been explored. There is a significant research lacuna in the way entomopathogen *T. asperellum* functions; its process of attachment into the outer cell of the cuticle of mosquito larvae and other pathogenicity processes. This research work takes up this research gap as its primary objective and explores further to gather knowledge about methods and mechanisms of novel entomopathogen *T. asperellum*. The consolidated objectives of this research work are as follows: (1) observation of host–pathogen interaction by compound light and Scanning Electron Microscopy, (2) detection of mucilage on *T. asperellum* spore surface at attachment site on larval surface, (3) detection of spore bound pr1 (Pathogenesis related 1 protein), chitinase and protease (caseinase) enzymatic activities of *T. asperellum,* iv) comparison of enzymatic activity of *T. asperellum* with known entomopathogens *i.e. Beauveria bassiana* (GenBank Accession No. KM604668.1) and *Metarhizium anisopliae*, observation of nuclear morphology of hemocytes cells of ME (methanolic extract) treated larvae and percentages of hemocyte degradation. Our study may provide the effective insights on mode of entomopathogenecity exhibited by *T. asperellum.*

## Results

### Mycoparasitism or host–pathogen interaction study through microscopy

**Figure 1:**
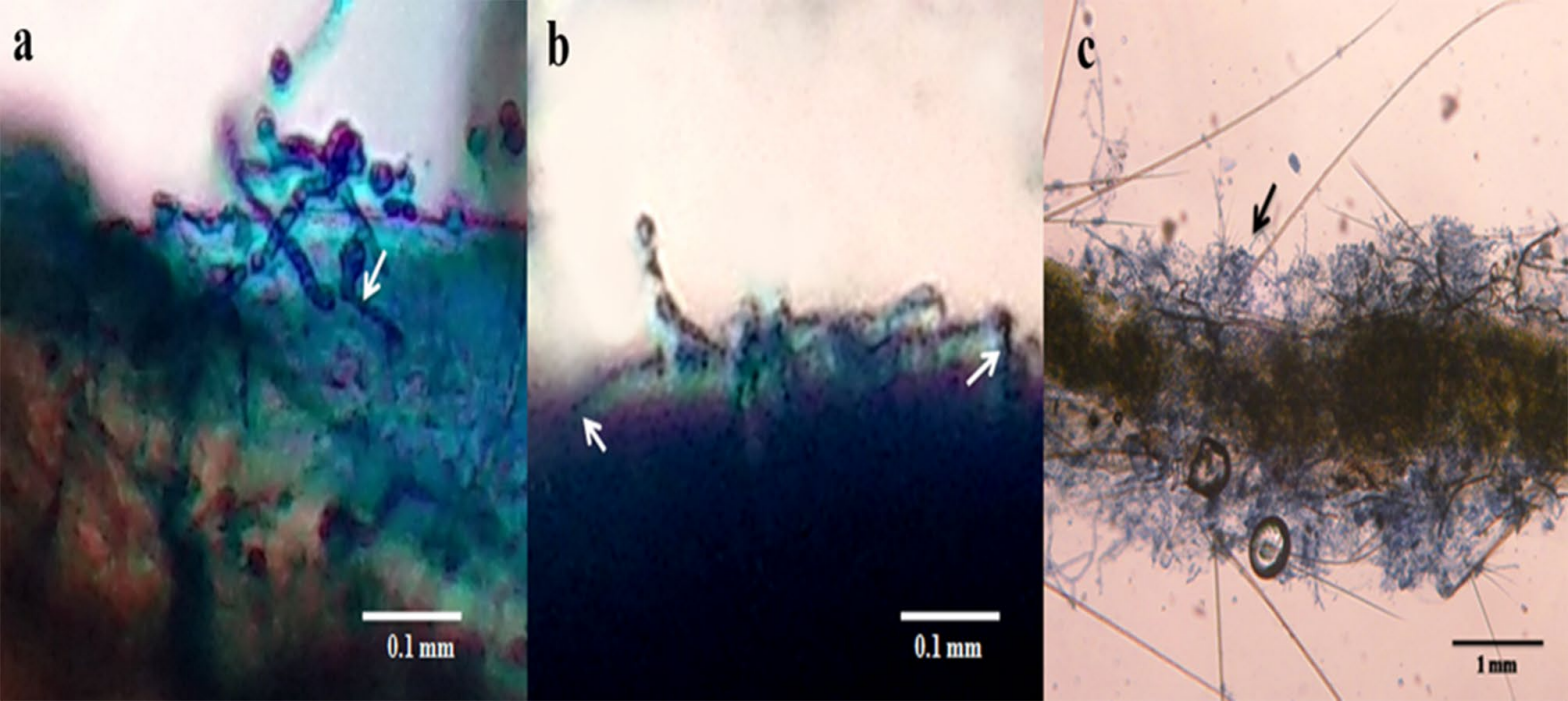
Interaction of *T. asperellum* spore with anopheline larvae under compound microscope (Olympus CX-31). (**a**) Germination of fungal spore and germ tube formation on larval surface (40×). (**b**) Cuticle penetration by formation of appresorium and infection peg (40×). (**c**) Hyphal proliferation of fungus on dead larvae (10×).

**Figure 2:**
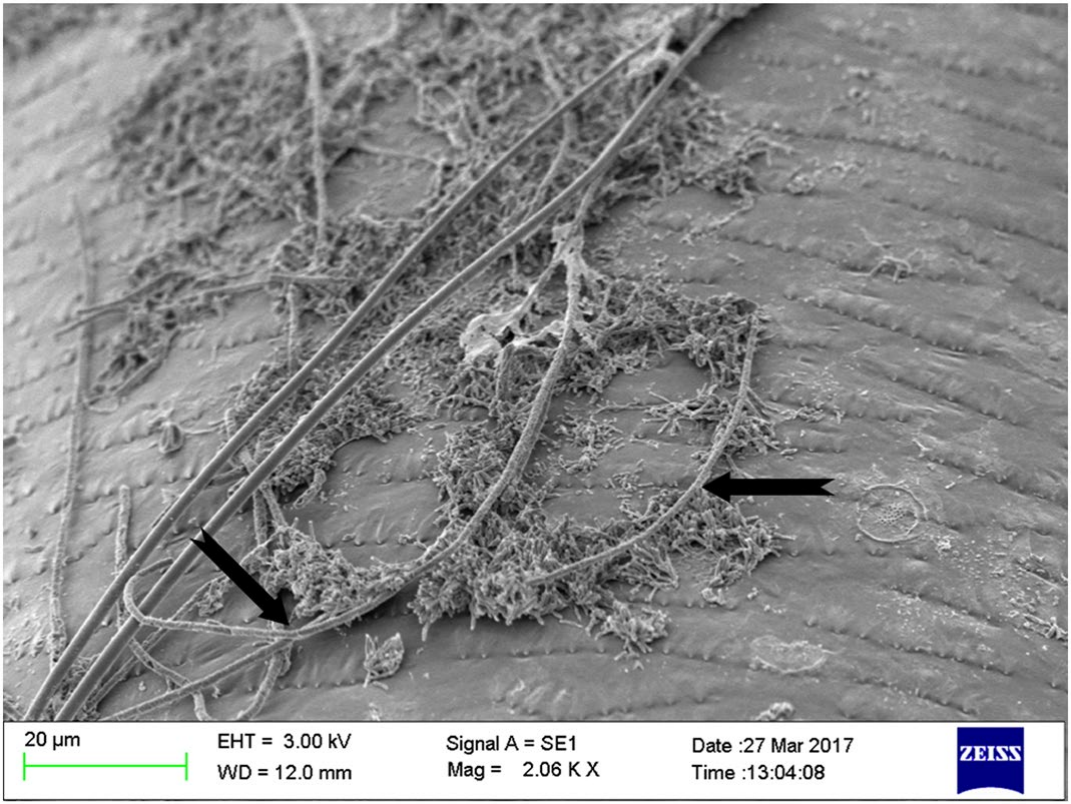
Scanning Electron Micrograph of proliferation of fungal hyphae on epicuticle of larvae (3rd instar) after 15 h of interaction. Portions of fungal hyphae indicated with black arrows.

Compound microscopic determination of lethality: Fungal spore treated mosquito larvae (3rd instars) were taken out at different times and stained with lactophenol cotton blue to observe host–pathogen interaction. After 5 h of interaction, spores were found to be germinated on larvae. Appresorium and infection peg were observed after 8 h of interaction. Proliferated hyphal growth on epicuticle of dead larvae was observed after 15 h of infection (Fig. 1). Scanning Electron Microscopic study revealed the hyphal proliferation on host epicuticle layer (Fig. 2) after 15 h of interaction.

### Spore adherence on host surface

**Figure 3:**
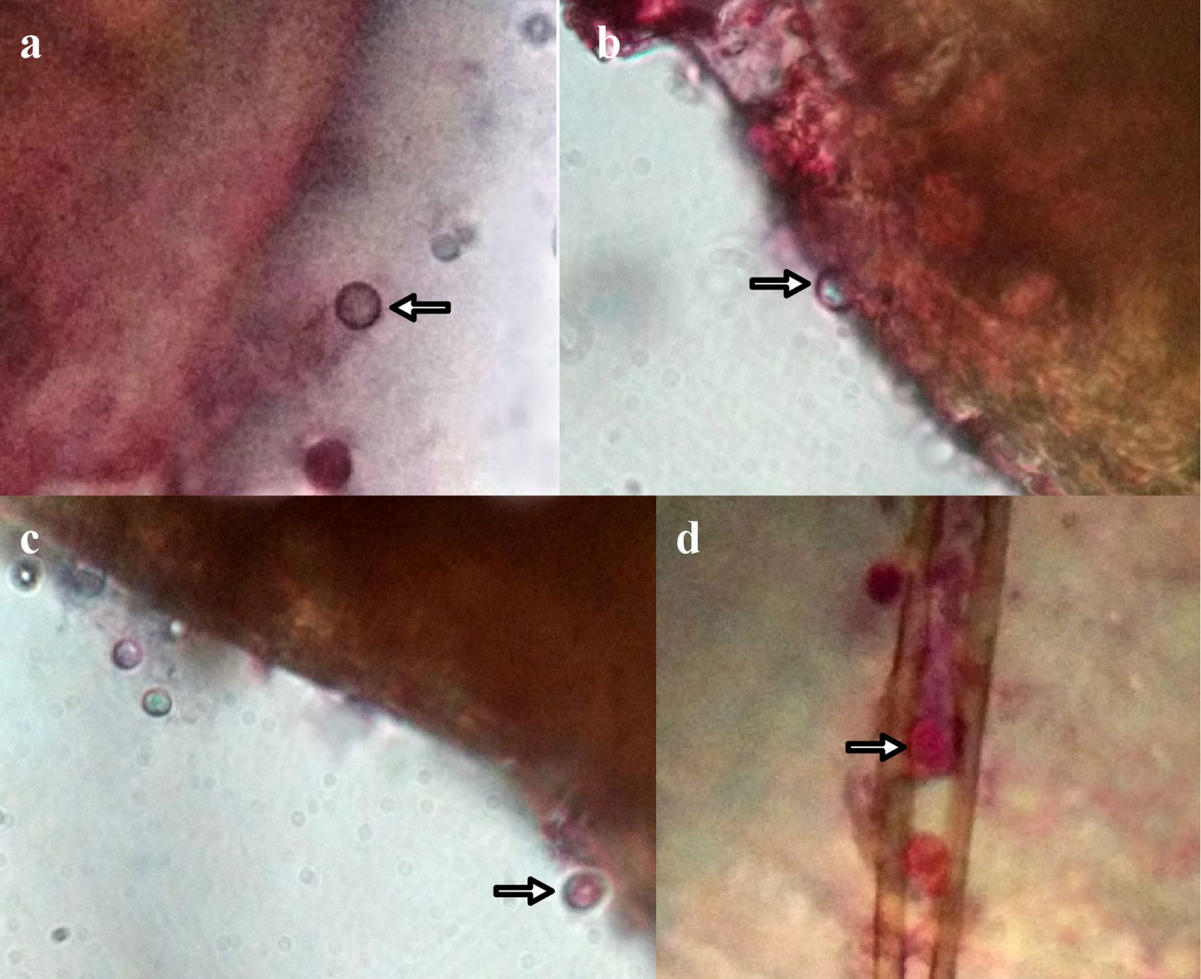
Adhesion of fungal spore on host surface under compound microscope (Olympus CX-31). (**a**, **b**, **c**) Fungal spore attachment on different surfaces of epicuticle of larvae, (**d**) attachment of spores on larval appendages, detected by mucilage specific dye (Ruthenium red).

Fungal spore treated mosquito larvae were taken out at different times and stained with Ruthenium red (0.1%) to detect the spore adhesion and the presence of mucilage during the attachment process. After 3 h of interaction, spores were found to be attached on larval surface exhibiting pink colored outer layer at the site of attachment (Fig. 3) indicating the presence of mucilage surrounding the attached spores.

### Production of hydrolytic enzymes by fungal pathogens

**Figure 4:**
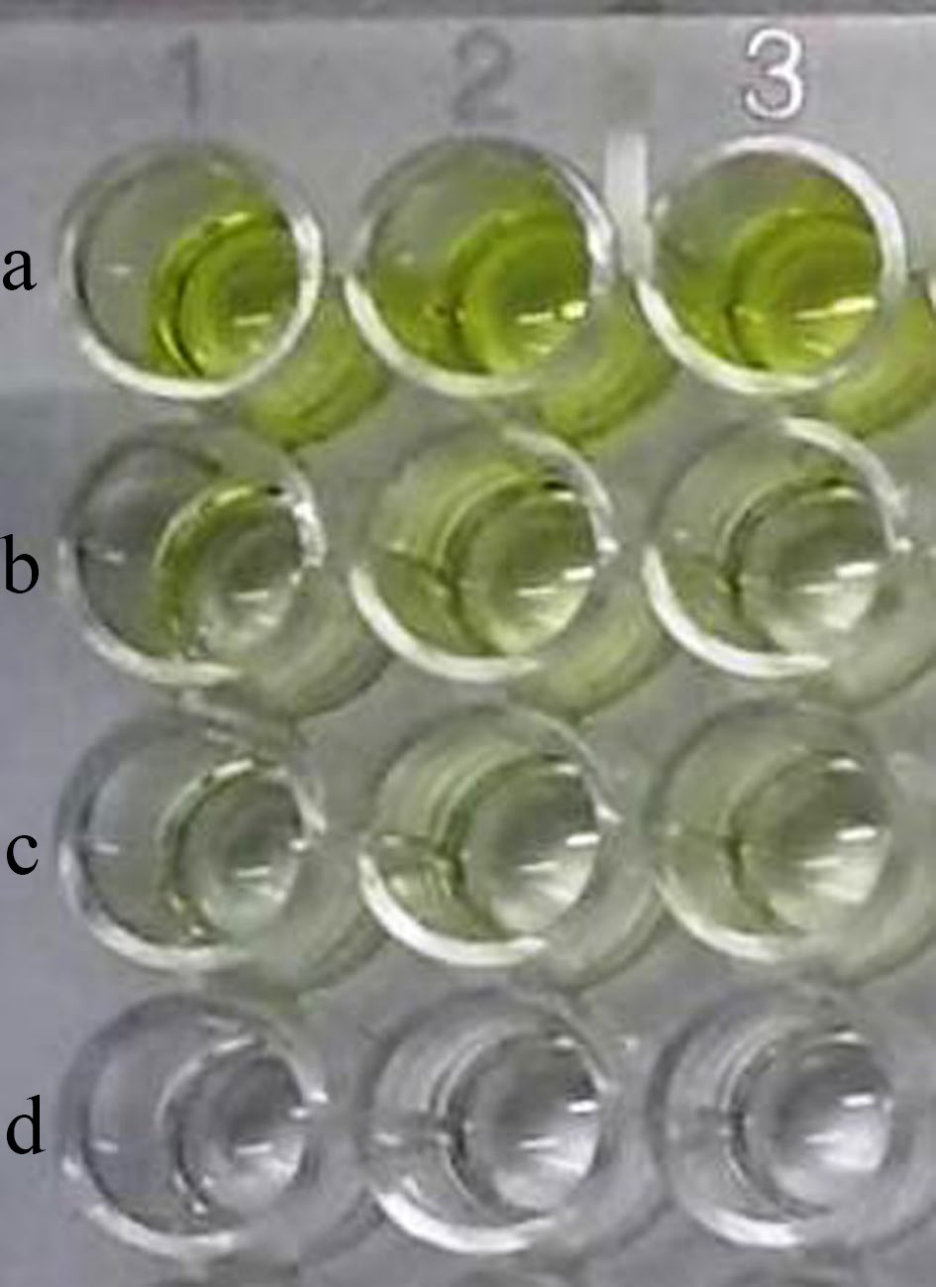
Formation of yellow coloured product (4-nitroaniline) after reaction of 4-nitroanilide with Pr1 protein, extracted from spore surfaces of three fungal isolates. (**a**) *T. asperellum*, (**b**) *B. bassiana*, (**c**) *M. anisopliae*, (**d**) control.

*Enzymatic assay for spore-bound Pr1* Freshly harvested conidia of fungal isolates inoculated with 0.1 M Tris–HCL supplemented with 1 mM succinyl-ala-ala-pro-phe-p-nitroanilide, exhibited yellow aqueous phase after reaction. The enzymatic cleavage of the 4-nitroanilide substrate yields 4-nitroaniline (yellow color under alkaline condition) which indicates the presence of spore-bound PR1 protein (Fig. 4). Absorbance was taken at 405 nm wave length and presented in the Table 1.

**Table 1:** Detection of hydrolytic enzymes (Spore bound PR1, Chitinase and Protease for different fungal isolates by spectro-photometric method.

Fungal isolates	Absorbance (OD value) in nm with mean ± SD)		
	Spore bound Pr1	Chitinase	Protease
*T. asperellum*	1.298 ± 0.002	0.899 ± 0.008	0.092 ± 0.004
*M. anisopliae*	1.171 ± 0.009	0.282 ± 0.007	0.089 ± 0.003
*B. bassiana*	0.565 ± 0.001	0.202 ± 0.007	0.090 ± 0.001
Control	0.103 ± 0.001	0.064 ± 0.002	0.061 ± 0.001

Data were expressed as mean ± standard deviation. The values were.

statistically significant at *p* < 0.05.

**Figure 5:**
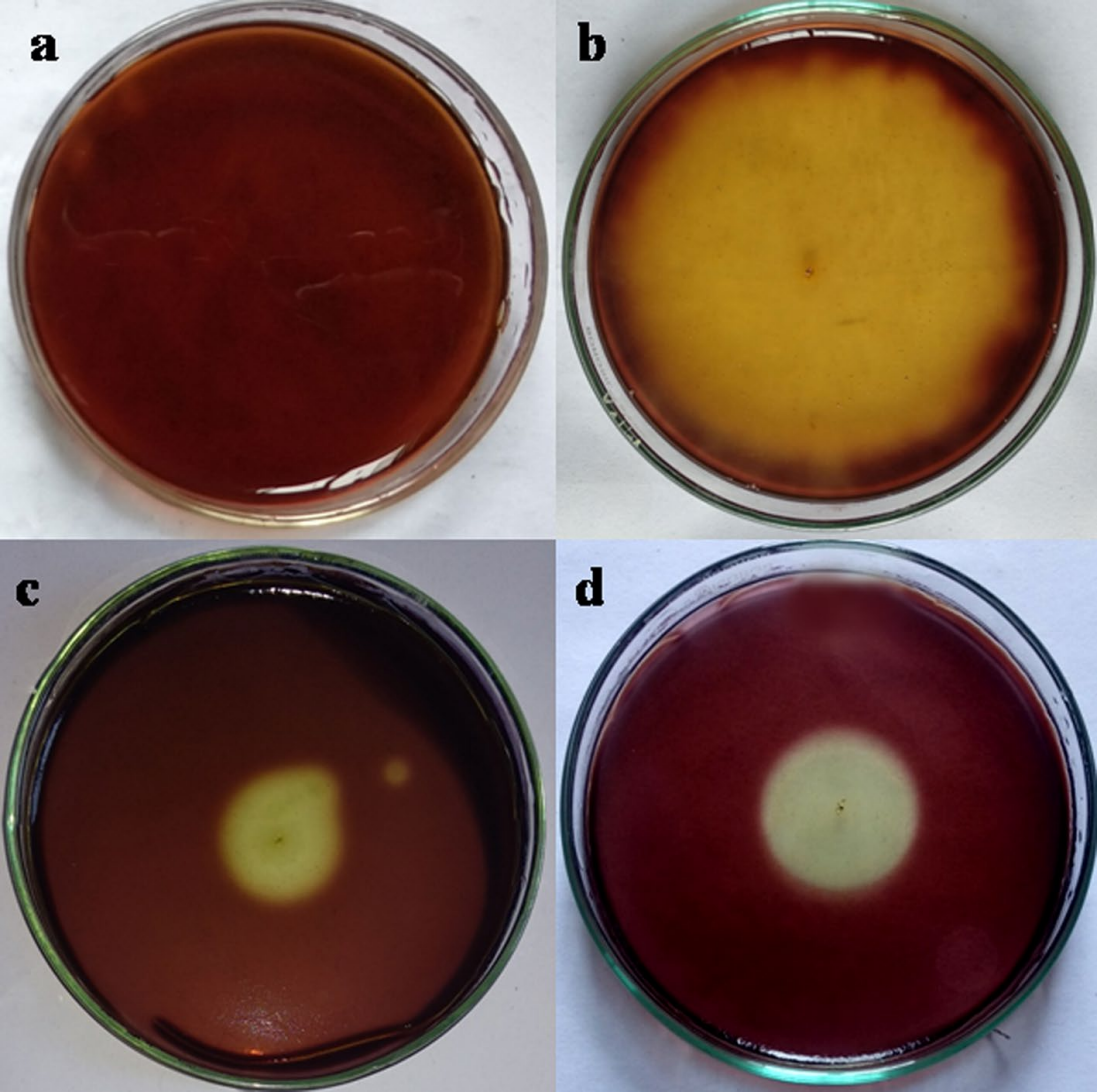
Hydrolytic zone of chitinase enzyme produced by three fungal isolates in chitin amended agar plates. (**a**) Control, (**b**) *T. asperellum,* (**c**) *B. bassiana*, (**d**) *M. anisopliae.*

**Figure 6:**
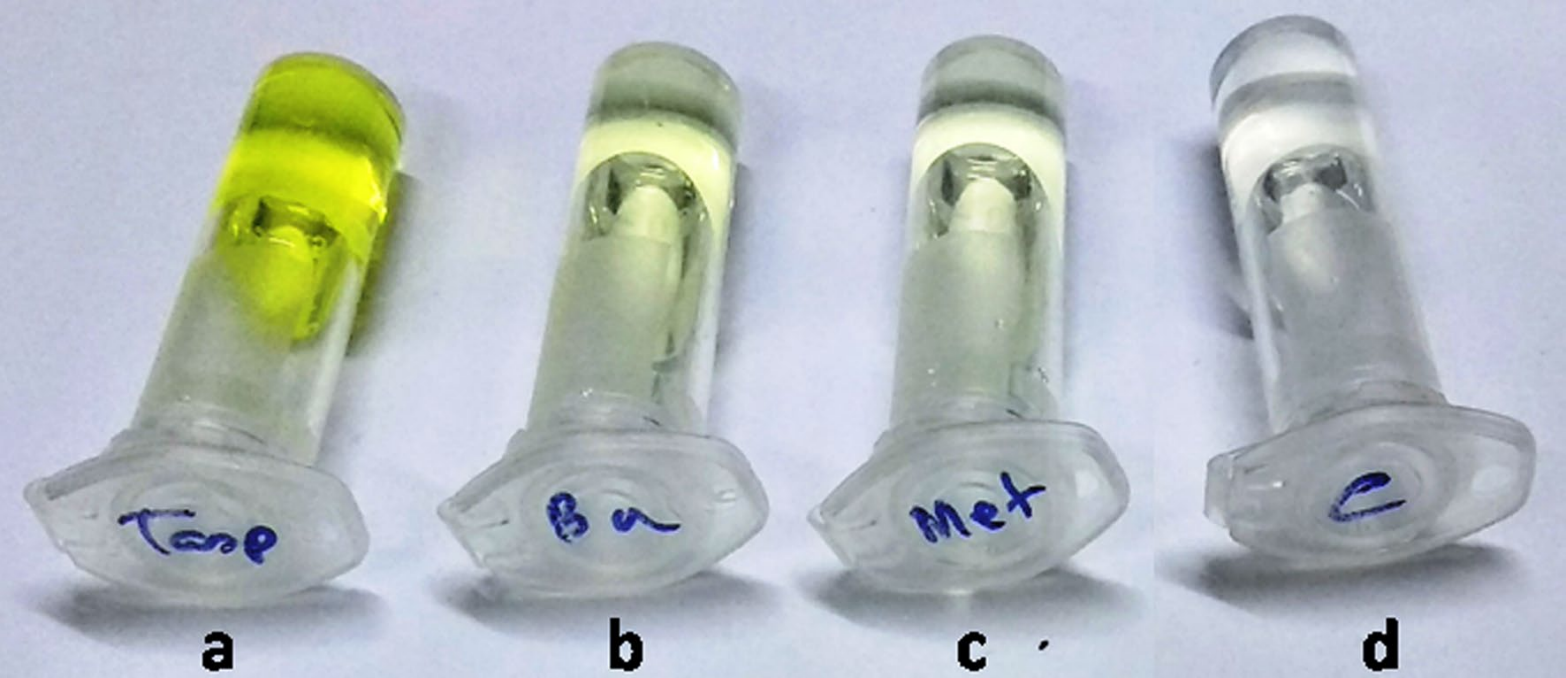
Formation of yellow coloured product (p-nitrophenol) after reaction of PNG with chitinase enzyme, extracted from culture filtrates of three fungal isolates. (**a**) *T. asperellum*, (**b**) *B. bassiana*, (**c**) *M. anisopliae*, (**d**) control.

*Chitinase detection* (1) *Detection of chitinase by plate assay method*: Preselected fungal isolates showed distinct enzyme hydrolytic zone in chitin amended Czapek Dox agar plates after five days of inoculation (Fig. 5). Relative enzymatic index (REI) was calculated for each fungus and presented in the Table 2. Hydrolytic zone diameter (8.4 cm) was noted best by *T. asperellum* among three entomopathogens. *T. asperellum* exhibited highest enzymatic index value (5.20) among three (Table 2). REI of *T. asperellum* was statistically same(*p* < 0.05) to *M. anisopliae* but different from *B. bassiana* (2) *Enzymatic assay for chitinase*: Fungal culture filtrate of fully grown preselected fungi (*T. asperellum*, *B. bassiana* and *M. anisopliae*) collected from chitin amended Czapek Dox broth were subjected to enzyme substrate reaction by adding PNG as substrate (Fig. 6). Absorbance was taken for each sample and OD values were presented in the Table 1.

**Table 2:** Relative Enzymatic Index (REI) of hydrolytic enzymes (chitinase and protease) of *T. asperellum*, *M. anisopliae* and *B. bassiana* in chitin and caesin amended agar plates.

Fungal isolates	Hydrolytic zone diameter (cm) (Mean ± SD)		Colony diameter (cm) (Mean ± SD)		REI	
	Chitnase	Protease	Chitin amended plate	Casein amended plate	Chitnase	Protease
*T. asperellum*	8.4 ± 0.68	8. 5 ± 1.78	2.0 ± 0.17	4.8 ± 1.56	5.20a	2.77a
*M. anisopliae*	3.2 ± 0.38	1.4 ± 0.11	0.78 ± 0.03	0.8 ± 0.013	5.10a	2.75a
*B. bassiana*	2.5 ± 0.11	0.6 ± 0.02	0.7 ± 0.002	0.4 ± 0.01	4.57b	2.5a

Data were expressed as mean ± standard deviation. The values were statistically significant at *p* < 0.05. Different letters in different rows indicate that they are statistically different as per Duncan analysis (*p* < 0.05).

**Figure 7:**
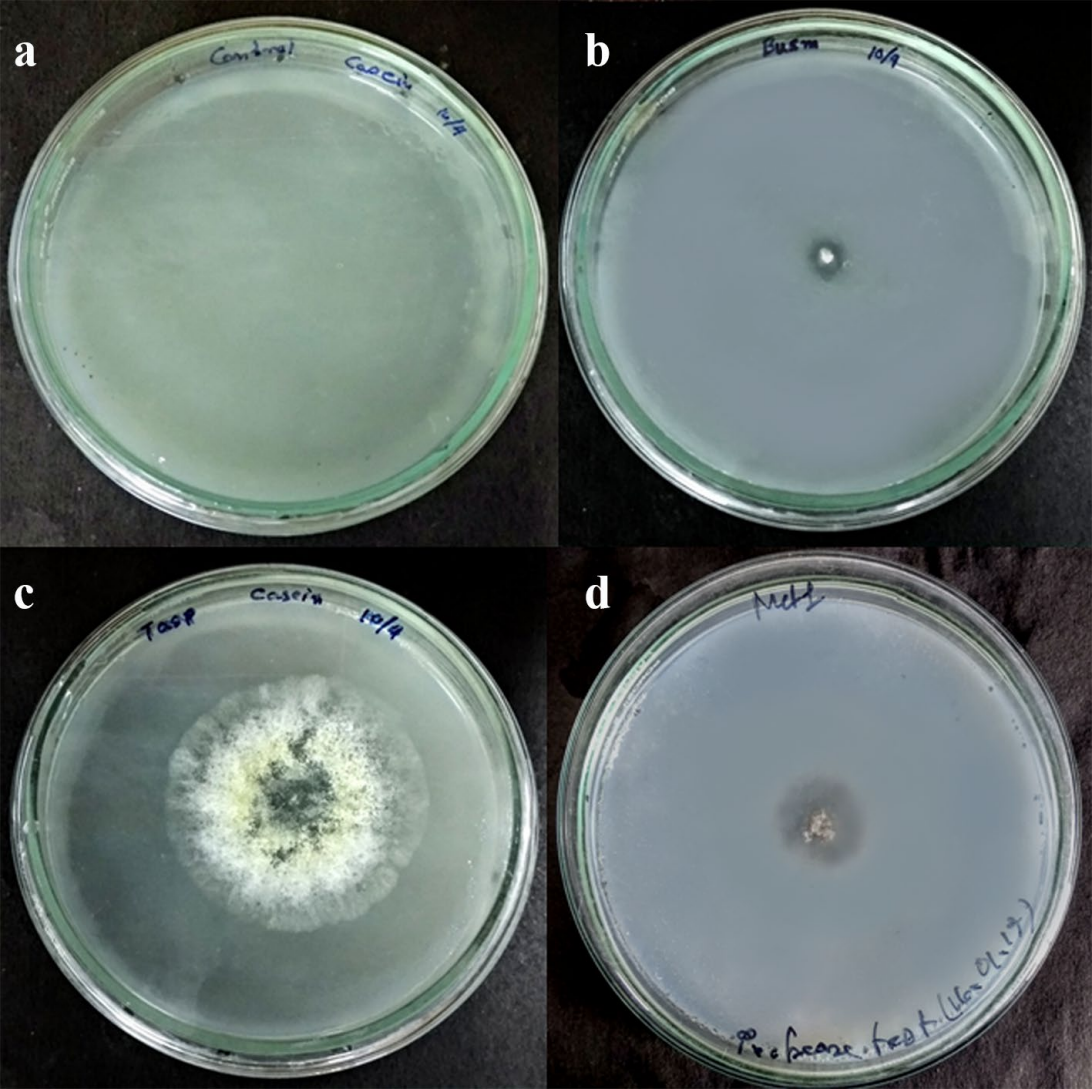
Hydrolytic zone of protease enzyme produced by three fungal isolates in casein amended agar plates. (**a**) Control, (**b**) *B. bassiana*, (**c**) *T. asperellum*, (**d**) *M. anisopliae.*

*Protease Detection* (1) *Detection of protease by plate assay method*: Preselected fungal isolates (*T. asperellum, M. anisopliae*, *B. bassiana*) exhibited distinct enzyme hydrolytic zone in casein amended Czapek Dox agar plates after five days of inoculation (Fig. 7). Relative enzymatic index was calculated for each fungus and presented in the Table 2. *T. asperellum* exhibited highest enzymatic index value which is 2.77 amongst three entomopathogens but statistically (*p* < 0.05) same to other two entomopathogens. (2) *Enzymatic assay for Protease*: Fungal culture filtrates of fully grown preselected fungi (*T. asperellum*, *B. bassiana* and *M. anisopliae*) collected from casein amended Czapek Dox broth were subjected to enzyme substrate reaction by adding casein powder as substrate. Absorbance was taken for each sample and OD values were presented in the Table 1.

**Figure 8:**
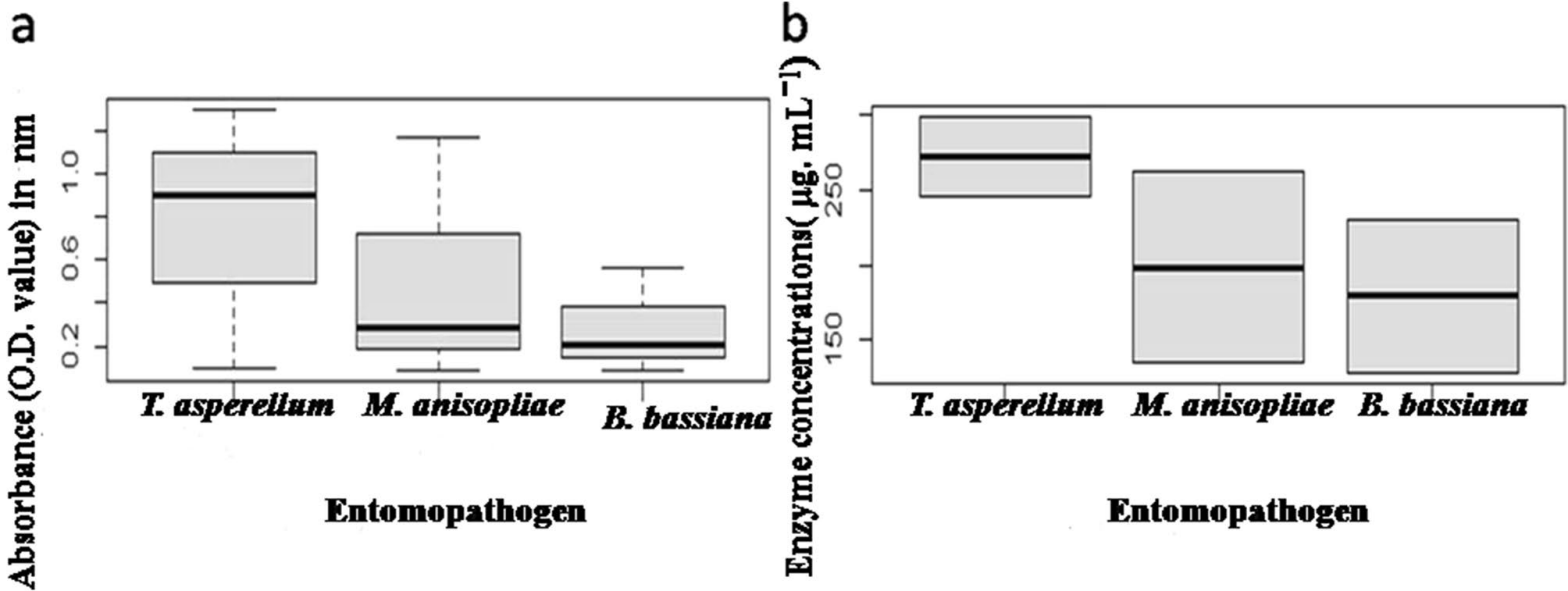
Boxplot representing the comparison of absorbances (OD values) of enzymatic assays and hydrolytic enzyme concentrations of three entomopathogens.

The t-test indicated t = 3.2181, df = 8, *p* value = 0.01227. The t-test (*p* value 0.01) showed the mean ≠ 0, so we can say that there are significant mean differences among the fungal pathogens for the OD values of hydrolytic enzymes. Box plot model (Fig. 8) proved that *T. asperellum* is the most enzymatically (Chitinase, Protease and Spore bound PR1) active pathogen among the three.

### Quantitative estimation of chitinase and protease enzymes

**Figure 9:**
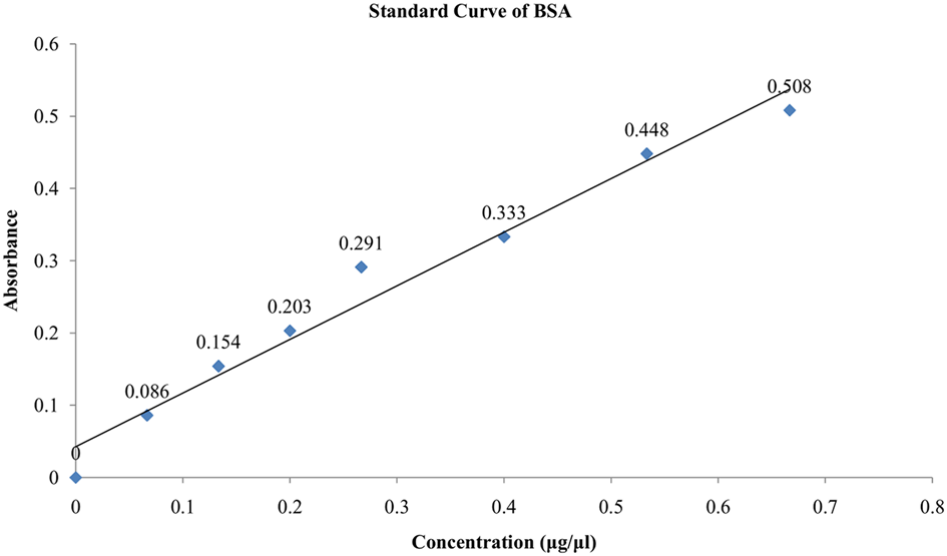
Standard curve of different known concentrations of BSA.

*Chitinase estimation* The estimated concentrations of chitinases from culture filtrates of different isolates were calculated using standard curve, constructed with absorbance of different known concentrations of BSA (Fig. 9). The chitinase concentrations of three isolates were presented in the Table 3. The data (Table 3) showed that chitinase enzyme concentration of *T. asperellum* was 245.204 ± 30.4 µg mL^−1^, whereas for *M. anisopliae,* it was 134.598 ± 26.6 µg mL^−1^ and for *B. bassiana*, it was 128.659 ± 22.78 µg mL^−1^ (*p* < 0.05) . They are statistically different as per Duncan analysis.

**Table 3:** Absorbances and concentrations of chitinase and protease enzymes in culture filtrate of three isolates.

Fungal isolates	Absorbance (OD value) in nm (Mean ± SD)		Concentration (µg mL^−1^) of enzyme (Mean ± SD)	
	Chitinase	Protease	Chitinase	Protease
*T. asperellum*	0.273 ± 0.006	0.345 ± 0.033	245.204 ± 30.4a	298.652 ± 54.09a
*M. anisopliae*	0.116 ± 0.003	0.297 ± 0.055	134.598 ± 26.6b	263.02 ± 51.56b
*B. bassiana*	0.124 ± 0.016	0.253 ± 0.054	128.659 ± 22.78c	230.358 ± 42.43c

Data were expressed as mean ± standard deviation. The values were.

statistically significant at *p* < 0.05. Different letters in different rows indicate that they are statistically different as per Duncan analysis (*p* < 0.05).

*Protease estimation* The estimated concentrations of proteases from culture filtrates of different isolates were calculated using standard curve, constructed with absorbances of different known concentrations of BSA (Fig. 9). The protease concentrations of three isolates were presented in the Table 3. The data (Table 3) showed that protease enzyme concentration of *T. asperellum* was 298.652 ± 54.09 µg mL^−1^) whereas *M. anisopliae* exhibited the value of 263.02 ± 51.56 µg mL^−1^ for the same and for *B. bassiana*, it was 230.358 ± 42.43 µg mL^−1^ (*p* < 0.05). They are statistically different as per Duncan analysis. Moreover, the t-test indicated t = 7.6075, df = 5, *p* value = 0.00006235. The t-test (*p* value 0.0006) showed the mean ≠ 0, so we can say that there are significant mean differences among the fungal pathogens in secreting different mean cumulative concentrations of hydrolytic enzymes (chitinase and protease). Box plot model (Fig. 8) proved that *T. asperellum* secretes the most concentration of hydrolytic enzymes among the three.

### Determination of nuclear deformities of treated larval hemocytes

**Figure 10:**
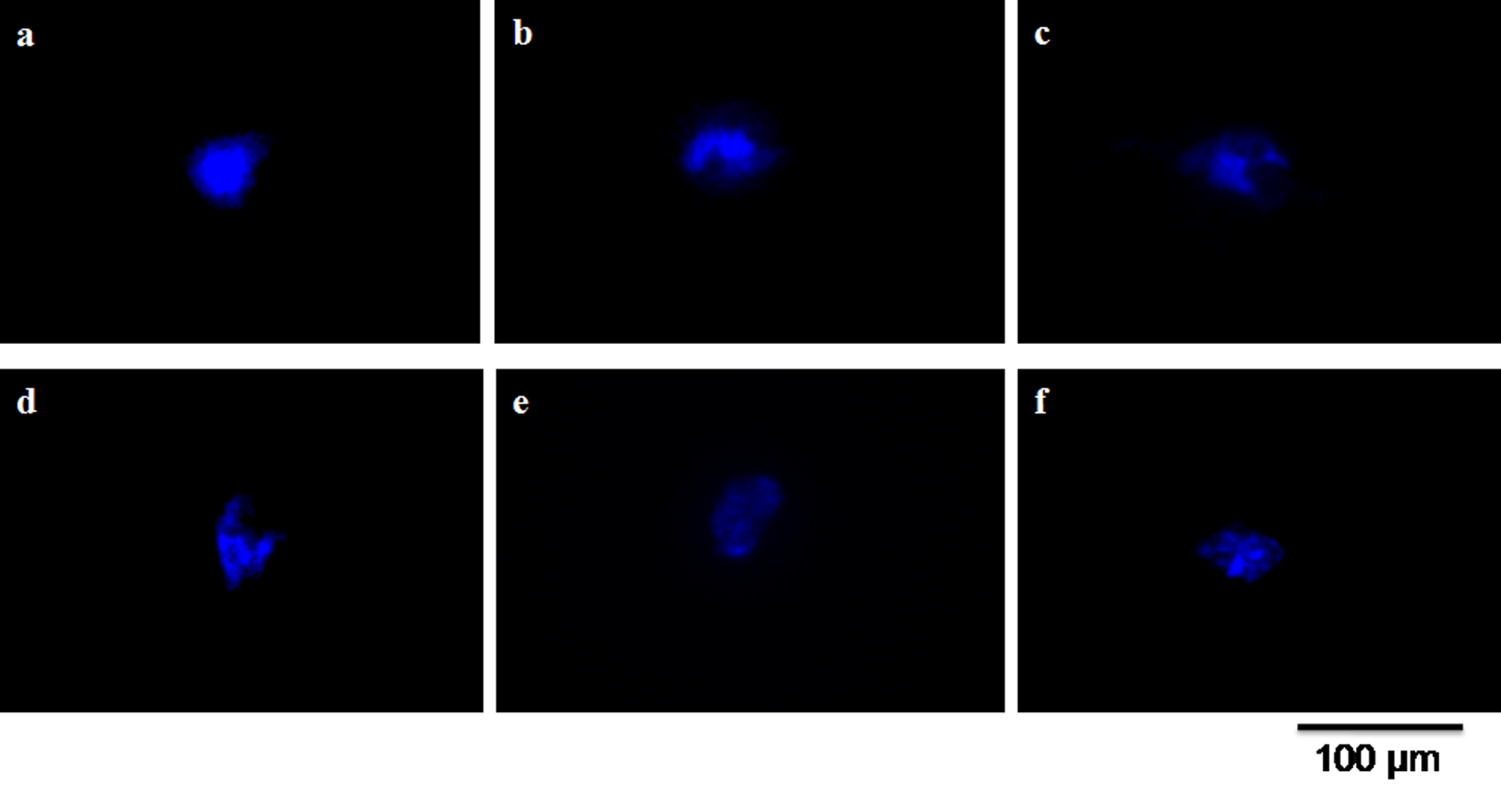
Fluorescent micrographs of nuclear morphologies of larval hemocytes. Hemocytic nucleus of (**a**) non-treated (control), (**b**) 0.02 mg mL^−1^ of ME treated, (**c**) 0.04 mg mL^−1^ of ME treated, (**d**) 0.06 mg mL^−1^ of ME treated, (**e**) 0.08 mg mL^−1^ of ME treated and (**f**) 0.1 mg mL^−1^ of ME treated larvae.

DAPI (4′,6-diamidino-2-phenylindole) stained, treated (ME of different doses) mosquito larval hemocytes and control nuclei of larva were presented in Fig. 10a–f. The treated (ME of different doses) mosquito larval hemocytes exhibited increased nuclear fragmentations in dose-dependent way (Fig. 10b–f). Degenerative nuclei were observed from different doses of ME treated larval hemocytes, compared with control which evinced normal spherical shaped nucleus (Fig. 10a). Mean percentage of number of hemocytes with nuclear deformities grew with increasing doses in comparison with control (Table 4). Mosquito larvae exposed to 0.1 mg mL^−1^ dose of ME showed maximum nuclear fragmentations and 100% of hemocytes exhibited nuclear deformities in this dose. 20, 45, 70 and 85% of nuclear deformities were recorded at 0.02, 0.04, 0.06 and 0.08 mg mL^−1^ concentration of ME. Result of Duncan analysis to measure significant mean differences was presented in Table 4. Different letters in different rows of the table indicate that they are statistically different as per Duncan analysis (*p* < 0.05).

**Table 4:** Percentage of deformed nuclear hemocytes at different ME doses of *T. asperellum*.

Concentration of ME (mg mL^−1^)	Percentage (%) of hemocytes with deformed nuclei
0.02	20e
0.04	45d
0.06	70c
0.08	85b
0.1	100a
Control	10f

Different letters in different rows indicate that they are statistically different as per Duncan analysis (*p* < 0.05).

### Proposed model of the fungi-larvae interaction

**Figure 11:**
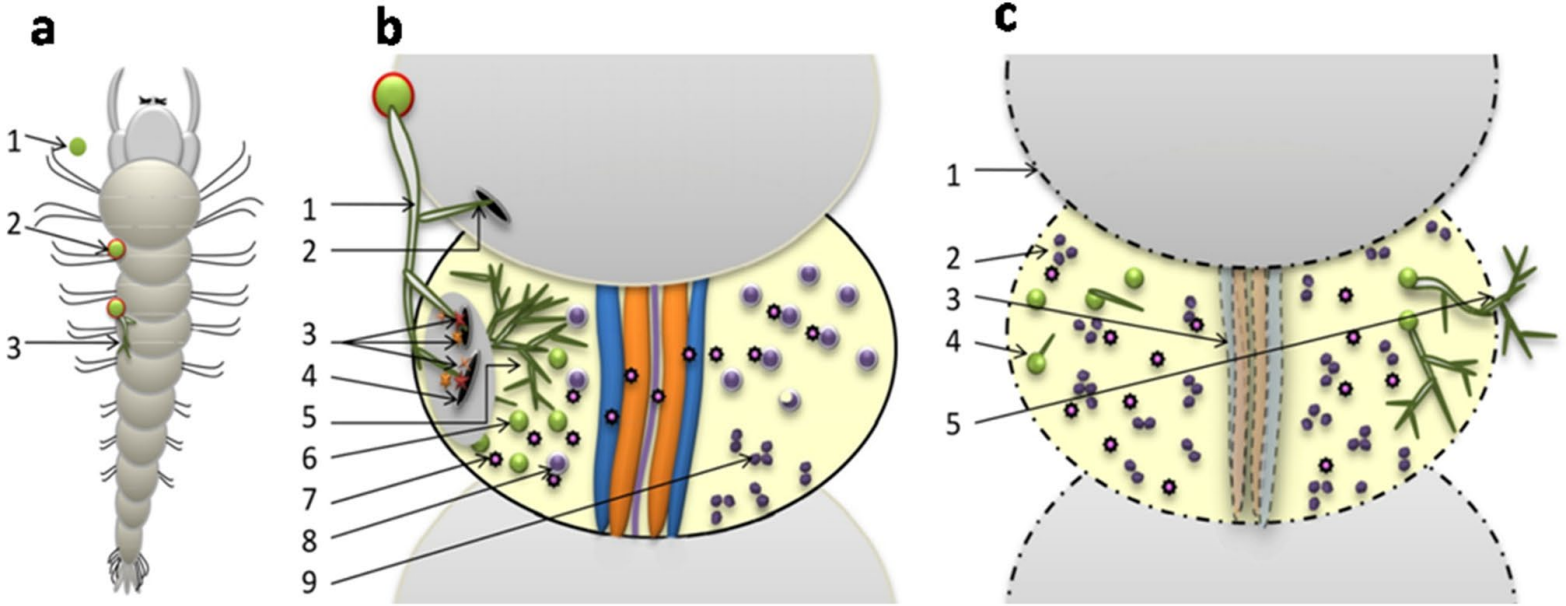
Schematic outline of fungi-larvae interaction. (**a**) Initial stages of fungal interaction, 1. Fungal spore before attachment, 2. Attachment of fungal spore on larval surface by mucilage secretion, 3. Germination of spore on host surface (**b**) Post germination stages of interaction, 1. Appresorrium formation, 2. Formation of infection peg, 3. Secretion of enzymes, 4. Larval cuticle degradation by enzymatic action, 5. Hyphal proliferation, 6. Sporulation, 7. Secretion of toxin, 8. Interaction of toxin with larval hemocyte, 9. Degeneration of hemocyte nucleus (**c**) Final stages of fungal interaction, 1. Degraded segment of dead larvae, 2. Degenerated hemocyte nuclei (immunity breakdown), 3. Shrinkage and deformation of alimentary system (based on information of our earlier work^11^), 4. Germination of spore inside dead larvae, 5. Proliferation of hyphae outside larval cuticle.

The proposed model of mechanism of entomopathogenic actions of *T. asperellum* on anopheline larvae was schematically outlined in Fig. 11.

## Discussion

In this present study, insect killing mechanism of a novel entomopathogen *Trichoderma asperellum* has been established and concentrations of secreted cuticle degrading enzymes of *T. asperellum* were compared with two known entomopathogens which were *B. bassiana* and *M. anisopliae*^19,20^. To establish a successful attachment to the host surface, fungi have to secrete adhesives containing carbohydrates with protein moiety^12^. Previous study supports that spore tip mucilage is responsible for attachment of fungal spore to the host surface^21^. Mechanism of spore adhesion on larval surface is determined by detecting the presence of mucilage at spore attachment site on host surface, using mucilage specific dye, Ruthenium Red^22^. Our study also validates that this fungus adapts to secret mucilage on spore wall for host attachment. Hyphal growth of the fungus on cuticle of mosquito larvae is observed by compound microscopy and SEM. To invade the insect cuticle, which is mainly composed of chitin and several other proteins, entomopathogens must secrete chitinase and protease enzymes^23^. Mechanism of enzymatic degradation of larval cuticle for hyphal penetrance by this fungus inside larval body is confirmed by the detection of spore bound pr1, chitinase and protease enzymes which are secreted by the fungi. In enzymatic assay, *T. asperellum* exhibits highest absorbance in end product of enzyme–substrate reaction confirming the presence of spore bound pr1 in appropriate amount, chitinase and protease with highest quantity amongst the three entemopathogens. In agar plate assay for protease and chitinase, highest relative enzymatic index is also recorded for *T. asperellum*. Highest concentrations of chitinases and protease enzymes are exhibited by *T. asperellum* amongst the three entemopathogens in quantitative protein estimation. The larva generally respond to the fungal infection by humoral mechanisms. After hyphal penetrance through larval cuticle, fungi secrete toxins, inside the larval body, which deteriorate larval immunity to complete the infection process^24^. Phenol oxidase (PO) of insect hemolymph and cuticle generally acts as a part of innate immunity of insect against infecting microbes. Larval PO is associated with melanin biosynthesis and haemocyte production for self defense; decreased PO indicates reduction of immunity which provide favorable conditions for pathogens growth inside the insect’s body^25^. In our previous study, we have reported decreased larval phenol oxidase content in ME treated larval hemolymph and cuticle, in comparison with control^11^. For mycochemistry of ME , the crude ME was fractionized, and each fraction was evaluated against anopheline larvae^11^. MF8, out of 12, was most lethal to anopheline larvae. GC–MS analysis of MF8 confirmed us that 49 compounds were present. Out of these compounds, seven compounds were recorded as insecticidal or mosquitocidal in our previous work^11^. Hemocyte, circulating immune cell, which is a vital component of larval innate immunity, destroys fungal pathogens by phagocytosis^26^. In *Drosophila* larvae, hemocytes serve immunological protection by melanizing and engulfing microbes and producing antimicrobial peptides. In addition, these immune cells by phagocytosis scavenge the apoptotic cells and play an important role during metamorphosis of this fly^27–29^. Here we have detected fragmented nuclei of hemocyte cells by fluorescence microscopy in treated larvae with different ME doses of *T. asperellum,* and also observed increased percentage of hemocyte degeneration in dose-dependent manner. Our observation reveals that the mode of action of the fungus weakens larval immunity resulting in the larval death. Furthermore, statistical analysis of data from enzymatic assays of *T. asperellum*, *B. bassiana* and *M. anisopliae* exhibits that data were statistically significant (*p* < 0.05) and there are significant different among the three entomopathogens in respect to hydrolytic enzyme secretion and we found that *T. asperellum* secretes highest concentrations of cuticle degrading (hydrolytic) enzymes amongst these three entemopathogens. In our previous study^11^ we have showed that *T. asperellum* has lower LD_50_ (lethal dose for 50% killing of larvae) and LT_50_ (lethal time for 50% killing of larvae) value amongst other known entomopathogens. Mechanistic study and comparison of enzymatic assay with other two also validate that *T asperellum* can be more effective than other two well known entomopathogens in controlling mosquito larvae.

After meticulous examination in the laboratory this research work comes to a fruitful conclusion which would contribute to future research works in this specific field. The mechanistic study of *T. asperellum* exhibits following mode of infections to kill anopheline larvae: (1) Secretion of mucilage from spore for its attachment on larval surface, (2) After germination of spore, penetration of insect cuticle by secretion of cuticle degrading enzymes (spore bound Pr1, chitinase and proteases) through infection peg, and (3) After penetration, secretion of toxins inside larval body which decrease larval phenol oxidase and degenerate hemocyte cells by nuclear fragmentations causing larval immunity breakdown followed by death. Application of the fungus as a new effective bio-control agent to eradicate anopheline larvae can open up new direction of *Trichoderma* research as entomopathogen and play a major role in mosquito vector control and disease management programme.

## Materials and methods

### Isolation of fungi and their identification

In this work fungal entomopathogens such as *T. asperellum (*Accession No. MG719999.1). *Beauveria bassiana* (Accession No. KM604668.1) and *Metarhizium anisopliae* were used which were previously isolated and identified (Phenotypically and PCR based ITSs of r DNA) in our laboratory. *T. asperellum*. and *M. anisopliae* from soil of Khardaha (North 24 Parganas), and *B. bassiana* from larva of insect in Kaliachak (Malda) and the procedures for isolation and identification were described in our previous published work^11^.

### Study of mycoparasitism or host parasitic interaction through microscopy

Compound microscopic study of host–pathogen interaction: Mosquito larvae (3rd instar), treated with LD_50_ dose (Lethal dose for 50% killing of larvae) (2.68 × 10^7^ conidia / mL) of *T. asperellum* (LD_50_ was determined by regression analysis of the bio- assay result in our previous study) ^11^ were taken out from the treatment set with needle and stained with lactophenol cotton blue solution in a grease free slide and mounted with cover-slip. Slide was observed under compound microscope to detect mosquito-fungi (host- pathogen) interaction. Scanning electron microscopic study (SEM study) of host–pathogen interaction was also conducted. *T. asperellum* spore treated infected mosquito larvae were subjected to SEM as demonstrated by Campos et al^30^.

### Detection of fungal spore adhesion on larval surface (Mucilage assay)

Twenty anopheline larvae (3rd instar), were exposed to LD_50_ dose (2.68 × 10^7^ conidia / mL) of *T. asperellum* spore as estimated in our early paper^11^. Larvae were taken from treatment set at each 1 h of interval in grease free slides to examine the fungal spore adhesion on larval surface. 100 µL of 0.1% Ruthidium Red (Mucilage specific stain^22^ was applied to each slide to detect the presence of mucilage on spore surface at spore attachment site on larval cuticle under compound microscope. Presence of mucilage can be detected by alteration of red color of the dye into pink^31^.

### Detection of cuticle degrading enzymes (Spore bound pr1, Chitinase and Protease)

#### Spore-bound Pr1 enzyme assay

Spore bound Pr1 activities of preselected fungi (*T. asperellum*, *B. bassiana* and *M. anisopliae*) were assayed following the modified protocol of Shah et al.^32^. Fungal entomopathogens were grown in PDA plates and ten milligrams of conidia were harvested after definite incubation period. Conidia of each fungus were inoculated in 1 mL of 0.1 M Tris–HCL supplemented with 1 mM succinyl-ala-ala-pro-phe-p-nitroanilide (C_30_H_36_N_6_O_9_) (Sigma-Aldrich) and incubated for 5 min at room temperature (28 ± 1 °C). After incubation, centrifugation of the sample was done at 12,000 g for 10 min at 4 °C. The yellow aqueous phase was collected after separating conidia from the sample and transferred to wells in a flat-bottom microtiter plate. Absorbance was taken at 405 nm using ELISA (Microtiter) reader (Biorad, USA). Experiments were done in triplicates for each fungus. Buffer substrate was used as control for each set.

### Chitinase and protease enzyme assay

#### Detection of chitinase and protease by plate assay method

##### Preparation of colloidal chitin, growth medium and inoculation

To prepare colloidal chitin from chitin flakes, modified protocol of Hsu and Lockwood^33^ was followed. In a 100 mL beaker, 20 mL HCl was taken and 1 g of chitin flakes was added slowly into it in the ratio of 20:1 and placed in a state of continuous stirring overnight at 4 °C upon a magnetic stirrer. After overnight stirring, the entire solution was added to 400 mL of cold distilled water (20 volumes) under continuous stirring. Chitin flakes were converted into colloidal form in this process. Then the solution was centrifuged at 2000 rpm for 15 min at 4 °C and supernatant was discarded. Thus, the precipitate obtained, was colloidal chitin which was highly acidic. The colloidal chitin was washed with cold distilled water repeatedly until the final pH become 7.0. In a 1000 mL beaker, 500 mL of Czapek Dox agar medium (NaNo_3_: 1 g; KH_2_PO_4_: 0.5 g; MgSO_4_, 7H_2_O: 0.25 g; KCl: 0.25 g; FeSO_4_, 7H_2_O: 0.05 g; Agar: 10 g) was prepared. 1% of colloidal chitin (5 mL) was suspended to it. The medium was sterilized by autoclave and poured in petridishes under sterile condition in Laminar Airflow Chamber. *T. asperellum*, *B. bassiana* and *M. anisopliae* were inoculated separately in plates containing the Czapek Dox agar media with colloidal chitin under sterile condition by hyphal tip inoculation method and incubated in B.O.D at 28 ± 2 °C. For each entomopathogen five replica of plates were taken.

##### Preparation of Czapek Dox agar medium with casein powder and inoculation

Czapek Dox agar medium was prepared and sterilized. 1% (w/v) of casein powder was mixed in the medium after the medium became little cold, and poured into the petridishes. Modified protocol of Parida et al.^34^ was followed. *B. bassiana*, *M. anisopliae* and *T. asperellum* were inoculated separately in plates containing the Czapek Dox agar medium with casein powder under sterile condition by hyphal tip inoculation method and incubated in B.O.D at 27 ± 2 °C. For each entomopathogen five replica of plates were taken.

##### Enzyme hydrolytic zone detection

For detection of chitinase enzyme hydrolytic zone, after 3 d of inoculation, each petridish was flooded with Grams Iodine solution^35^. Grams iodine binds with soluble, unhydrolyzed chitin forming brown color complex. Chitinase activity was visualized by detecting clear hydrolytic zone surrounding the fungal colony. Clear zone was formed due to hydrolyzation of soluble chitin by the activity of chitinase enzyme where Grams iodine did not bind.

For detection of protease enzyme hydrolytic zone, 5 mL of 10% TCA after 5 d of fungal inoculation was pour in each plate containing the Czapek Dox agar medium with casein powder following the modified method of Medina and Baresi^36^ and Al Nahdi^37^.

Relative Enzymatic Index was determined with following formulae^34^ by calculating hydrolytic zone diameter and fungal colony diameter.

Relative Enzymatic Index (REI):

$$\frac{Zone\;diameter + Colony\;diameter}{{Colony\;diameter}}$$

#### Detection of chitinase and protease in broth culture

##### Preparation of medium and inoculation of fungal strain

Czapek Dox broth and colloidal chitin were prepared as described above. For chitinase assay, 1% of colloidal chitin was added to it following modified protocol of Murthy and Bleakley^38^ and for protease assay casein powder (1%) (w/v) was added into the medium after little cooling. For both cases 20 mL medium was taken in each 50 mL conical flask. *B. bassiana*, *M. anisopliae* and *T. asperellum* were inoculated separately in the Czapek Dox broth medium amended with colloidal chitin and also Czapek Dox broth medium fortified with casein under sterile condition and incubated in Shaker B.O.D at 150 rpm at 30 ± 2 °C for 5 d.

##### Enzymatic assay for chitinase and protease

To assay chitinase enzyme activity standard protocol was followed^39,40^. Chitinase produces p-nitrophenol (yellow color compound) in reaction with PNG. In a 96 well microtitre plate, 10 µL of fungal culture filtrate was taken from each set. 10 µL of 10 mM PNG (p-nitrophenyl β-D-N glucosaminide) and 30 µL of 0.1 M PBS (pH: 6) were added into it. Here PNG was used as substrate. Six replicas were taken for each fungus. The micro titer plate was incubated at 37 °C for 1 h. After that, reactions were stopped by adding 50 µL of Na_2_CO_3_ in each well. Absorbance was recorded at 415 nm wave length.

To determine protease activity, modified procedure of Tsuchida et al.^41^ was followed. After 5 d of inoculation 200 µL of culture filtrate was taken out from each fungal culture in separate eppendroff tube (2 mL). 500 µL of 1%( W/V) casein powder suspended in 50 mM PBS were added as substrate for each set. Then each set was incubated for 15 min in water bath at 45 °C temperature for enzyme substrate reaction. Then 1 mL of 10% TCA was added in each set to terminate the reaction. After that, each reaction mixture was centrifuged at 10,000 rpm for 15 min. 500 µL of supernatant was taken from each set in separate eppendroff tube. Then the supernatant was mixed with 1 mL of 0.4 M Na_2_Co_3_ and 0.5 mL of threefold diluted Folin-ciocalteu reagent and incubated at room temperature (37 °C) in the dark for 30 min. 100 µL of each resulting solution was taken in a 96 well microtitre plate, and absorbance of developing blue color was measured at 660 nm against reagent blank using distilled water in place of culture filtrate.

### Quantitative estimation of chitinase and protease enzymes

*Chitinase quantification.* Fungi were inoculated in 50 mL of 1% chitin amended media (as described before) and incubated at shaker B.O.D at 150 rpm and 28 ± 2 °C temperature. After 5 d of inoculation, 5 mL of culture filtrates were taken from the media and the total proteins were recovered by Acetone precipitation^42^ (sample: Acetone = 1:2) in 15 mL falcons. The precipitated proteins were subjected to quantitative estimation. A standard curve was prepared with different known concentrations of BSA by spectrophotometric method^43^. Concentration of chitinase enzymes were calculated using different known concentrations of BSA as standard by MS EXCEL, 2007.

*Protease quantification* Fungi were inoculated in 50 mL of 1% casein amended media (as described before) and incubated at shaker B.O.D at 150 rpm and 28 ± 2 °C temperature until the colour of casein disappeared. After that 5 mL of culture filtrates were taken from the media and the total proteins were precipitated by Acetone cut^42^ (sample: Acetone = 1:2) in 15 mL falcons. The precipitated proteins were quantified as described above.

### Staining of nucleus of larval haemocytes and fluorescence microscopy

The Cell suspension was placed in a grease free slide and 10 µL of aqueous DAPI solution (1 µg mL^−1^) were added^44–46^. The solutions were mixed properly by pipetting. Solution mixture were covered with a cover slip and incubated in dark for 15 min. After that, the slides were placed under inverted fluorescence microscope (Olympus-CKX53) for observation using Q imaging software.

### Statistical analysis

In each experiment mean and **±** SD (Standard deviation) of data were calculated.

In addition, in case of detection of chitinase and protease in broth culture experiment different OD value for each fungus was obtained during enzymatic assays. A t-test was performed using R (Ver: 4.1.0) with combining the OD values for enzymatic assays of each fungus to analyze whether the significant mean difference was present or not. A box plot model was also represented graphically using R (Ver: 4.1.0) for further confirmation. In case of quantitative estimation of chitinase and protease enzyme assay different mean concentrations of hydrolytic enzymes for each fungus (Chitinase and Protease) were obtained. A t-test was performed using R (Ver: 4.1.0) with combining the values for hydrolytic enzymes’ concentrations of each fungus to analyze whether the significant mean difference was present or not and also a Duncan analysis was performed to measure specific differences among means concentration of hydrolytic enzymes and REI of three entomopathogen. A Duncan analysis was also done to measure mean percentages of deformed nuclei of treated anopheline larvae using SPSS (Ver: 13.0).

## Data Availability

Data are available from www.nature.com.
